# Deletion of the *SKO1* Gene in a *hog1* Mutant Reverts Virulence in *Candida albicans*

**DOI:** 10.3390/jof5040107

**Published:** 2019-11-15

**Authors:** Verónica Urrialde, Daniel Prieto, Susana Hidalgo-Vico, Elvira Román, Jesús Pla, Rebeca Alonso-Monge

**Affiliations:** Departamento de Microbiología y Parasitología, Área de Microbiología, Facultad de Farmacia, Instituto Ramón y Cajal de Investigaciones Sanitarias (IRYCIS), Universidad Complutense de Madrid, Plaza de Ramón y Cajal s/n, E-28040 Madrid, Spain; veronicaurrialde@gmail.com (V.U.); adprieto@ucm.es (D.P.); shvico@ucm.es (S.H.-V.); elvirarg@ucm.es (E.R.); jesuspla@farm.ucm.es (J.P.)

**Keywords:** *Candida albicans*, virulence, stress response, transcription factor, commensalism

## Abstract

*Candida albicans* displays the ability to adapt to a wide variety of environmental conditions, triggering signaling pathways and transcriptional regulation. Sko1 is a transcription factor that was previously involved in early hypoxic response, cell wall remodeling, and stress response. In the present work, the role of *sko1* mutant in *in viv*o and *ex vivo* studies was explored. The *sko1* mutant behaved as its parental wild type strain regarding the ability to colonize murine intestinal tract, *ex vivo* adhesion to murine gut epithelium, or systemic virulence. These observations suggest that Sko1 is expendable during commensalism or pathogenesis. Nevertheless, the study of the *hog1 sko1* double mutant showed unexpected phenotypes. Previous researches reported that the deletion of the *HOG1* gene led to avirulent *C. albicans* mutant cell, which was, therefore, unable to establish as a commensal in a gastrointestinal murine model. Here, we show that the deletion of *sko1* in a *hog1* background reverted the virulence of the *hog1* mutant in a systemic infection model in *Galleria mellonella* larvae and slightly improved the ability to colonize the murine gut in a commensalism animal model compared to the *hog1* mutant. These results indicate that Sko1 acts as a repressor of virulence related genes, concluding that Sko1 plays a relevant role during commensalism and systemic infection.

## 1. Introduction

Living cells respond to external stimuli through signal transduction pathways. Surrounding changes are perceived by sensors that trigger specific signals through different pathways to transcription factors, which control gene expression. The signal transduction pathways that are mediated by MAP (Mitogen Activated Protein) Kinases are mechanisms that are conserved in all eukaryotic cells that play an important role in cell physiology and the response to stress [1]. In pathogenic microorganisms, these signaling pathways allow cells to face the host immune system [2]. The opportunistic pathogen *C. albicans* is a fungus that inhabits as a commensal in skin and mucosa of human beings, but behaves as a pathogen when immune barriers become broken or in immunocompromised individuals [3,4]. The HOG pathway, mediated by the MAP kinase Hog1, becomes activated in response to a wide variety of stresses [5,6,7,8,9]. Mutants in this pathway display enhanced sensitivity to oxidative agents, hyperosmotic conditions, metalloids, etc. Remarkably, the *hog1* mutant becomes avirulent in a systemic infection model in mice and is more susceptible to killing by phagocytes [10,11]. The *hog1* mutant is also unable to establish as a commensal in a murine gastrointestinal colonization model [12]. The *hog1* mutant is also more sensitive to Histatin 5 (Hst5), a cationic peptide that is present in the saliva with antifungal activity [13]. Hst5 triggers an osmotic stress response, increasing the expression, among other genes, of the transcription factor *SKO1*. Sko1 is the only transcription factor that has been reported to be phosphorylated by Hog1 upon osmotic stress [14] and probably by other stresses [15] in *C. albicans*. Transcriptomic analyses revealed that Sko1 controls the expression of genes that are implicated in carbohydrate metabolism, redox metabolism, and glycerol synthesis [16]. Other studies report that Sko1 regulates the early hypoxic response [17], although the *sko1* mutant grows in the absence of O_2_ similarly to the wild type and it is not sensitive to inhibitors of the electron transport chain contrary to what occurs to the *hog1* mutant [18]. Transcriptional studies suggest that Sko1 acts as a repressor [15], similarly to its homolog in *Saccharomyces cerevisiae* [19,20]. The deletion of the *SKO1* gene in a *hog1* defective mutant partially suppresses its osmosensitivity in *C. albicans* [15]. Moreover, Sko1 also acts as a transcriptional activator; this role has been extensively characterized in *S. cerevisiae*. In this microorganism, the phosphorylation of Hog1 upon osmotic or oxidative stress triggers the phosphorylation of Sko1. The phosphorylated form of Sko1 binds to Cyc8, Tup1, SAGA, and Swi/Snf, generating a complex at the target gene promoters, which activates or represses specific gene transcription [20,21,22].

In the present work, the role of Sko1 in commensalism and virulence was explored in *C. albicans*; the *sko1* mutant behaved as wild type in both murine commensalism and *Galleria mellonella* systemic infection models. Nevertheless, significant differences were observed when a double *hog1 sko1* double mutant was analyzed, suggesting that Sko1 controls the expression of genes during host-pathogen interaction.

## 2. Materials and Methods

### 2.1. Strains and Growth Conditions

Yeast strains that were used are listed in Table 1. To label the *C. albicans* strains, pNIM1R-GFP and pNIN1R-dTOM2 plasmids carrying, respectively, the GFP and dTOM2 fluorescent proteins under the control of the repressible tetracycline promoter *OP4* [12] were digested with *Kpn*I- *Ksp*I to force recombination at the *ADH1 locus*, generating strains RM100-GFP, *sko1*-dTOM2, and *hog1 sko1*-dTOM2, which express the fluorescent proteins constitutively in the absence of doxycycline.

Yeast strains were routinely grown at 37 °C in YPD medium (1% yeast extract, 2% peptone, and 2% glucose). SD medium (2% glucose, 0.67% yeast nitrogen base) plus amino acids and chloramphenicol (10 mg/L) was used to distinguish *C. albicans* strains that were labelled with dTOM2 from strains that were labelled with GFP.

The influence of the surrounding atmosphere was analyzed either by incubating the plates in an incubator that was designed for cell culture or in an anaerobic chamber. The cell culture incubator was programmed at 37 °C, 80% humidity, and 5% CO_2_ in the presence of atmospheric O_2_. Hypoxia was achieved using an anaerobic chamber and a commercial system, which ensures less than 0.1% O_2_ in 2.5 h and more than 15% CO_2_ (GENBox anaer, BioMérieux).

Drop tests were performed by spotting ten-fold serial dilutions of cells onto YPD plates that were supplemented with different concentrations of bile salts and incubated at 37 °C for 24 h.

### 2.2. Virulence Assays in Galleria mellonella

*C. albicans* cells that were grown overnight in YPD at 37 °C were collected and washed twice in phosphate buffer saline (PBS). The cells’ concentration was calculated by Neubauer chamber and 10^6^ cells in 10 µL were injected into the hemocoel at the last left pro-leg using a Hamilton syringe. Then, 20 larvae of approximately 400–500 mg weight were used for each infection group. Two groups were used as a control: larvae injected with PBS and larvae not inoculated. Larvae were maintained at 37 °C in darkness. Survival was monitored for 9 days after infection. Kaplan-Meier survival curves are shown and Log-rank (Mantel-Cox) test statistical analyses were performed.

### 2.3. Murine Intestinal Commensalism Model and Adhesion Assay

All experiments involving animals that were performed in this work were carried out in strict accordance with the regulations in the “Real Decreto 1201/2005, BOE 252” for the Care and Use of Laboratory Animals of the “Ministerio de la Presidencia”, Spain. The protocol that was used in the commensalism model was approved by the Animal Experimentation Committee of the University Complutense of Madrid (CEA 25/2012, BIO2012-31839-1) and Comunidad de Madrid according to Artículo 34 del RD 53/2013. All efforts were made to minimize suffering, even though the treatments did not result in disease in the animals. The number of animals that were used in the experimentation was minimized for ethical reasons. 

The protocol for studying commensal colonization, which was used in this work, has been described previously [12]. Briefly, after 7 days of antibiotic pre-treatment (2 mg/mL streptomycin, 1 mg/mL bacitracin, and 0.1 mg/mL gentamycin), 10^7^ *C. albicans* cells were inoculated in a single gavage. Stool samples were obtained every 2 to 4 days, were homogenized in PBS, and were cultured in SD plates to determine CFUs per gram. To analyze *C. albicans* loads in different portions of the gastrointestinal tract, mice were sacrificed and samples from the stomach, cecum, small and large intestine were aseptically separated, homogenized, and diluted in sterile PBS and cultured in SD plates.

To analyze the capacity to adhere to intestinal mucosa, we proceeded as previously described [12]. The Adhesion Relative Index was calculated by dividing the percentage of adhered cells from (wt or *sko1*)-dTOM2 strains, which were recovered by their percentage in the inoculum.

## 3. Results

### 3.1. The Lack of Sko1 Does not Alter the Ability to Colonize the Murine Intestine

**Figure 1:**
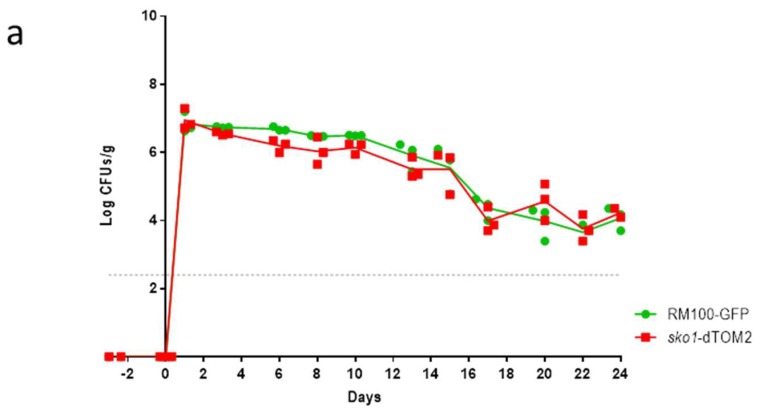

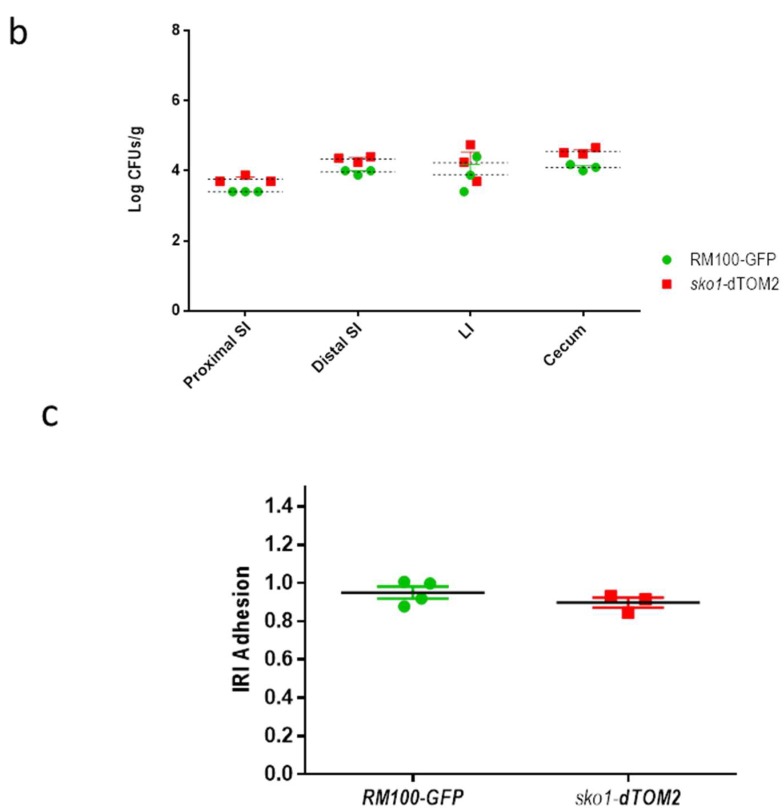
Role of Sko1 in murine gut colonization. **a**) C57BL/6J mice were treated with a combination of antibiotics for 1 week to reduce bacterial microbiota. Then, 10^7^ *C. albicans* cells in 100 µL of a balanced mix of *sko1*-dTOM2 and wt-GFP cells were inoculated intragastrically. The graph represents Log CFUs/ g of faeces across time. Each square represents a single mouse (*n* = 3); **b**) At day 24, mice were euthanized and their intestine was split on proximal small intestine (SI), distal small intestine, large intestine (LI), and cecum. Samples were processed and *C. albicans* colonies were counted. Each single independent value is represented as the mean ± SEM from three mice; **c**) Adhesion to intestinal mucosa was assayed using a mix of wt-GFP/wt-dTOM2 tagged strains as internal control and a mixture of wt-GFP and *sko1*-dTOM2 strains as samples. *C. albicans* mix was allowed to adhere to clean murine gut mucosa for 150 min and then the Adhesion Relative Index was calculated by dividing the percentage of adhered cells from wt or *sko1* strains labelled with the red fluorescence protein, which was recovered after 150 minutes of interaction with gut mucosa, by their percentage in the inoculum. Each point represents an individual assay.

Since Sko1 has been implicated in the early response to hypoxic growth conditions in *C. albicans* [17], we wonder if this transcription factor was required for *C. albicans* to establish as a commensal in the gastrointestinal tract due to its hypoxic environment. The *sko1* defective mutant was labelled with the dTOM2, a red fluorescence protein, while the parental strain RM100 was labelled with GFP. Then, a balanced mixed culture of both strains was intragastrically inoculated in mice that were previously treated with a combination of antibiotics. Colonization level was followed in time by CFUs, which were counted from stools (Figure 1a). Both strains colonized murine gut to the same level, displaying no significant difference between them. At the end of the experiment, the amount of each strain in the intestinal content of different portions of the intestine was quantified by the CFUs count (Figure 1b). The *sko1* mutant and the wild type strain showed a similar distribution along the intestine. 

Adhesion to murine intestinal mucosa was also analyzed. Both labelled strains were mixed to an equal amount and were left to adhere to small intestine tissue (obtained from mice that were not previously colonized) for 150 min (Figure 1c). Then, the relative adhesion index was calculated for both strains. All these results indicate that the lack of *sko1* neither altered the adhesion to intestinal mucosa nor the ability to colonize murine gut. 

### 3.2. The sko1 Mutant is More Resistant to Bile Salts

**Figure 2:**
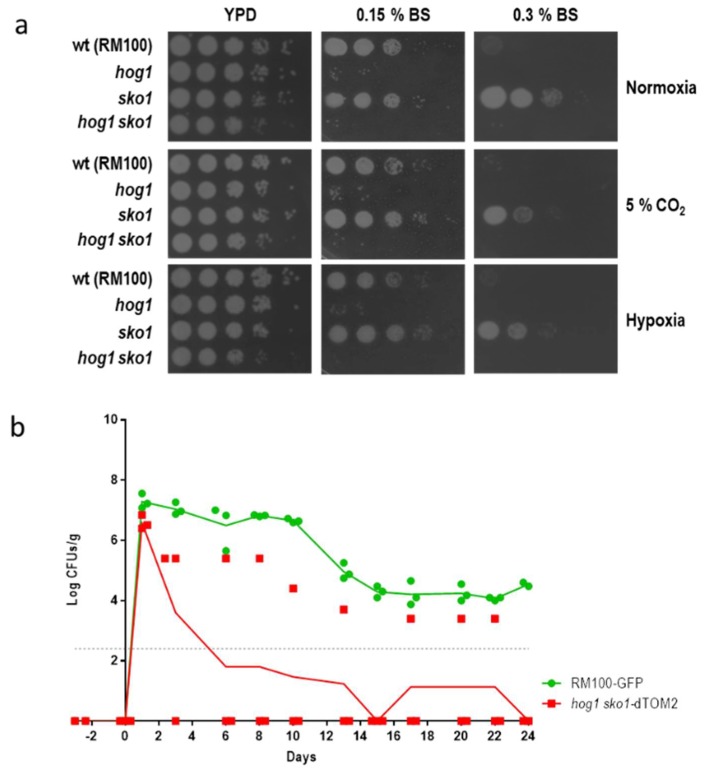
Influence of *SKO1* deletion in a *hog1* background. a) ten-fold dilution of cell suspensions of the indicated strains were spotted on YPD plates that were supplemented with different amounts of Bile Salts (BS). Plates were incubated under the specified conditions at 37 °C for 24 h (normoxia and 5% supplemented CO_2_ atmosphere) or 48 h (for plates incubated under hypoxia). b) C57BL/6J mice that were pre-treated with antibiotics were inoculated with a balanced mix of wt-GFP/*hog1 sko1*-dTOM2 cells and colonization was followed across time. The graph represents Log CFUs/ g of faeces versus time. Each square represents a single mouse (*n* = 3).

The susceptibility to bile salts was also texted by spotting cell suspensions on YPD plates that were supplemented with a commercial mixture of bile salts. The *sko1* mutant displayed a clear resistance to the presence of bile salts (Figure 2a). This resistance was evidenced at high bile salts concentration (0.3% BS) since under these conditions, the parental RM100 was not able to grow. Moreover, the resistance to bile salts was observed under different environmental conditions: normoxia, hypoxia, or normoxia plus 5% CO_2_, suggesting that this phenotype does not depend on the amount of O_2_ or CO_2_. Sensitivity to bile salts has been related to a defect in the colonization [12] or impaired colonization in the proximal small gut at early stages of the colonization [25]. We tested if this enhanced resistance to bile salts could be detected in the double *hog1 sko1* mutant and if this phenotype affects the ability to colonize murine gut. The *hog1 sko1* double mutant displayed a severe sensitivity to bile salts under all tested conditions, similar to the sensitivity that was displayed by the *hog1* mutant (Figure 2a). 

Likewise, the ability of the *hog1 sko1* double mutant to colonize the murine gut was also analyzed. This mutant displayed a colonization rate significantly reduced compared to the wild type strain (and the *sko1* mutant). Remarkably, in one of the three analyzed mice, the *hog1 sko1* mutant was able to colonize the gut to detectable levels (Figure 2b), indicating that deletion of the *SKO*1 gene in a *hog1* mutant strain improved survival into the murine gut. The *hog1* mutant was unable to establish gut colonization and was removed from the gut after 2 to 3 days post-inoculation [12].

### 3.3. Sko1 is not Required for Virulence, but Lack of Sko1 Enhances the Virulence of a hog1 Mutant

**Figure 3:**
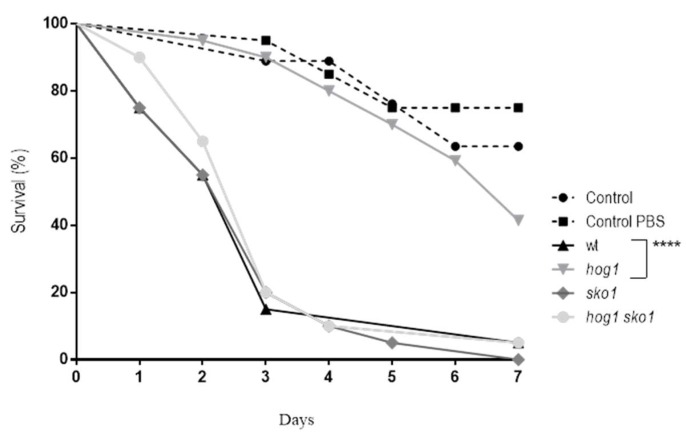
Virulence of *C. albicans* in a *G. mellonella* systemic infection model. 10^6^ *C. albicans* cells were injected at the last left pro-leg of *G. mellonella* larvae and survival of the insect was followed in time. Control refers to *G. mellonella* larvae that was not inoculated and control PBS indicates larvae that was inoculated with PBS as stress control. Kaplan-Meier survival curves are shown and the Log-rank (Mantel-Cox) test displayed significant differences between the *hog1* mutant and the parental wild type strain. **** *p* <0.0001.

Previous studies showed that the Sko1 transcription factor was not required for virulence in *C. albicans* [15]. These studies were performed using a systemic infection model in mice. In the present work, the virulence of the *sko1* mutant was analyzed using an alternative infection model: *Galleria mellonella* larvae. To perform this, virulence assays 1 × 10^6^ *C. albicans* cells were injected in the last pro-leg of *G. mellonella* larvae and viability was followed in time (Figure 3). As expected, the *sko1* mutant behaved similarly to the wild type strain, meanwhile the *hog1* mutant was not virulent in this animal model. Surprisingly, the *hog1 sko1* mutant behaved as the *sko1* mutant and wild type strains, reverting the virulence of the *hog1* mutant. 

## 4. Discussion

Metabolic adaptation and the ability to respond to environmental stresses have been extensively associated with *C. albicans* pathogenesis [26,27]. The capacity of *C. albicans* to sense changes in the environment and straight away respond, adapting to the new conditions as well as the capability to use a wide variety of nutrients, allows this fungus to inhabit different niches and cause diverse diseases. *C. albicans* is able to proliferate over the human body (skin and mucosa) besides its ability to disseminate to different organs, escaping from the immune system. Sko1 is a transcription factor that controls the expression of stress related and metabolic genes [16]. This transcription factor has been placed downstream of the Hog1 MAP kinase in response to osmostress and downstream of Psk1 in response to cell wall disturbing compounds [14]. Although no sensitivity to osmotic stress was detected in the *sko1* mutant at 1 M NaCl concentration [15], significant growth defect was detected at higher salt concentrations (1.5 M or 2 M NaCl) [16]. In addition, the *sko1* mutant displays a clear sensitivity to caspofungin and other cell wall disturbing compounds [14,15]. In spite of its role in osmotic stress signaling and cell wall biogenesis, the Sko1 transcription factor was dispensable for virulence in the murine systemic infection model and to survive phagocytes [15]. Here, we show that Sko1 is also not required for *C. albicans* to colonize murine gastrointestinal tract. Moreover, the distribution along the small and large intestine was similar in the *sko1* mutant and the wild type strain. There were no differences between wild type and *sko1* mutant strains regarding adhesion to intestinal epithelium *ex vivo*. Sko1 was also not required for virulence using the alternative invertebrate model *G. mellonella*. All these data suggest that Sko1 does not play a relevant role during systemic infection or commensalism in spite of its role in the stress response, cell wall biogenesis, and metabolism [15,16] .

Surprisingly, the *sko1* mutant displayed an increased resistance to bile salts. Bile salts play a role in the digestion and absorption of lipids from the diet and, remarkably, these compounds have antimicrobial activity as they disturb the membranes. The *sko1* mutant was more resistant to bile salts than the wild type strain; this resistance could compensate the defect in the *sko1* mutant cell wall and somehow allow the colonization of the gastrointestinal tract. There are two possibilities for why the *sko1* mutant is resistant to bile salts: a) the *sko1* displays a different membrane composition that is less susceptible to these compounds or b) bile salts cannot get the plasma membrane due to a different cell wall composition. Although no alteration on the membrane has been reported for the *sko1* mutant to our knowledge, a role as a repressor of fatty acid metabolism genes was reported by Marotta and co-workers [16]. Genes such as the fatty acid oxidase *FOX2* and the lipase *TGL1* are overexpressed in the *sko1* mutant. The *TGL1* gene remains uncharacterized in *C. albicans* and its ortholog codifies an enzyme with sterol esterase activity. Tgl1 is an integral component of the membrane, which function is to mobilize steryl ester stores from the membrane and to release free sterols and fatty acids in *S. cerevisiae* [28]. The overexpression of the *TGL1* gene that was observed in the *sko1* mutant may alter the membrane composition and therefore, alter the resistance to bile salts. The alterations in the membrane that confer resistance to bile salts are Hog1-dependent, suggesting that Hog1 is also involved in lipid homeostasis.

The role of Sko1 as a repressor of genes that are involved in the osmotic stress response has been previously reported in *C. albicans* [15,16]. The passage through the intestine could trigger an osmotic stress response in *C. albicans*, mainly in the large gut where the water is absorbed from the lumen. The lack of Sko1 in a *hog1* mutant background may allow the expression of genes that improve the osmotolerance *in vitro* [15] and, probably, the survival in the gut. The level of expression of these genes seems to not be enough to completely return the ability to become established as a commensal of the *hog1 sko1* double mutant, which is probably due to the fact that both genes, *SKO1* and *HOG1*, are required for the full expression of stress response genes [16].

Noteworthy, the deletion of the *SKO1* gene in a *hog1* mutant reverts the virulence and improves the ability to colonize the murine gastrointestinal tract. It was reported previously that Sko1 acts as transcriptional repressor of genes that are involved in virulence and filamentation [15]. Genes such as *ECE1*, *HWP1*, and *WH11* at a physiological temperature (37 °C) were upregulated in both *sko1* and *hog1* mutants, and the *hog1 sko1* double mutant displayed an even higher expression level of these genes [15]. The *ECE1* gene encoded for a hypha specific protein that became split on several peptides, among them, the peptide called candidalysin [29]. This peptide acted as a cytolytic toxin, damaging epithelial host membranes, leading to the release of damage associated cytokines. Further, *ece1* mutants were avirulent in the animal model of mucosal infection [30] as well as zebrafish and murine models of systemic fungal infections [31]. The upregulation of *ECE1* reported previously [15], together with other genes that were implicated in virulence, may explain the restoration of virulence in the *hog1 sko1* double mutant. This upregulation may not be enough to completely revert the ability of the *sko1 hog1* double mutant to colonize the murine gut. 

Although the *sko1* mutant behaves as the wild type strain concerning virulence and commensalism, the analyses of the *hog1 sko1* double mutant indicates that Sko1 mediates the expression of genes that are relevant for virulence and possibly for the commensal state of *C. albicans*. 

## Figures and Tables

**Table 1:** Strains that were used in this study.

Microorganism	Strain	Genotype	Nomenclature in the Manuscript and Figures	Source
*C. albicans*	RM100	ura3*Δ*::*imm*434/*ura*3Δ::*imm*434 his1Δ::*hisG*/*his1Δ*::*hisG-URA3-hisG*	wt	[23]
*C. albicans*	CNC13	ura3Δ::*imm*434/*ura*3Δ::*imm*434 his1Δ::*hisG*/*his1*Δ::*hisG* hog1::hisG-URA3-hisG/hog1::hisG	*hog1*	[24]
*C. albicans*	VIC100	ura3Δ*::imm434/ura3*Δ*::imm434* his1Δ*::hisG/his1*Δ*::hisG* sko1Δ*::hisG/sko1*Δ*::hisG-URA3-hisG*	*sko1*	[15]
*C. albicans*	VIC200	ura3Δ*::imm434/ura3*Δ*::imm434* his1Δ*::hisG/his1*Δ*::hisG* hog1::hisG/hog1::hisG sko1Δ*::hisG/sko1*Δ*::hisG-URA3-hisG*	*hog1 sko1*	[15]
*C. albicans*	RM-GFP	ura3Δ::*imm*434/*ura*3Δ::*imm*434 his1Δ::*hisG*/*his1*Δ::*hisG-URA3-hisG* ADH1/adh1::tTA pTET-*^Mo^GFP-SAT1*	RM100-GFP	This work
*C. albicans*	VIC101	ura3Δ*::imm434/ura3*Δ*::imm434* his1Δ*::hisG/his1*Δ*::hisG* sko1Δ*::hisG/sko1*Δ*::hisG-URA3-hisG* ADH1/adh1::tTA pTET-dTOM2-SAT1	*sko1*-dTOM2	This work
*C. albicans*	VIC201	ura3Δ*::imm434/ura3*Δ*::imm434* his1Δ*::hisG/his1*Δ*::hisG* hog1::hisG/hog1::hisG sko1Δ*::hisG/sko1*Δ*::hisG-URA3-hisG* ADH1/adh1::tTA pTET-dTOM2-SAT1	*hog1 sko1*-dTOM2	This work

## References

[1] Kultz D. (1998). Phylogenetic and functional classification of mitogen- and stress-activated protein kinases. J. Mol. Evol..

[2] Day A.M., Quinn J. (2019). Stress-Activated Protein Kinases in Human Fungal Pathogens. Front. Cell. Infect. Microbiol..

[3] Odds F.C. (1994). *Candida* species and virulence. ASM News.

[4] Odds F.C. (2010). Molecular phylogenetics and epidemiology of *Candida albicans*. Future Microbiol..

[5] Alonso-Monge R., Navarro-García F., Román E., Negredo A.I., Eisman B., Nombela C., Pla J. (2003). The Hog1 mitogen-activated protein kinase is essential in the oxidative stress response and chlamydospore formation in *Candida albicans*. Eukaryot. Cell.

[6] Urrialde V., Prieto D., Pla J., Alonso-Monge R. (2015). The Pho4 transcription factor mediates the response to arsenate and arsenite in Candida albicans. Front. Microbiol..

[7] Enjalbert B., Smith D.A., Cornell M.J., Alam I., Nicholls S., Brown A.J., Quinn J. (2006). Role of the Hog1 stress-activated protein kinase in the global transcriptional response to stress in the fungal pathogen *Candida albicans*. Mol. Biol. Cell.

[8] Cui S., Hassan R.Y., Heintz-Buschart A., Bilitewski U. (2016). Regulation of *Candida albicans* Interaction with Macrophages through the Activation of HOG Pathway by Genistein. Molecules.

[9] Herrero-de-Dios C., Day A.M., Tillmann A.T., Kastora S.L., Stead D., Salgado P.S., Quinn J., Brown A.J.P. (2018). Redox Regulation, Rather than Stress-Induced Phosphorylation, of a Hog1 Mitogen-Activated Protein Kinase Modulates Its Nitrosative-Stress-Specific Outputs. MBio.

[10] Alonso-Monge R., Navarro-García F., Molero G., Díez-Orejas R., Gustin M., Pla J., Sánchez M., Nombela C. (1999). Role of the mitogen-activated protein kinase Hog1p in morphogenesis and virulence of *Candida albicans*. J. Bacteriol..

[11] Arana D.M., Alonso-Monge R., Du C., Calderone R., Pla J. (2007). Differential susceptibility of mitogen-activated protein kinase pathway mutants to oxidative-mediated killing by phagocytes in the fungal pathogen *Candida albicans*. Cell. Microbiol..

[12] Prieto A.D., Román E., Correia I., Pla J. (2014). The HOG pathway is critical for the colonization of the mouse gastrointestinal tract by *Candida albicans*. PLoS ONE.

[13] Vylkova S., Jang W.S., Li W., Nayyar N., Edgerton M. (2007). Histatin 5 initiates osmotic stress response in *Candida albicans* via activation of the Hog1 mitogen-activated protein kinase pathway. Eukaryot. Cell.

[14] Rauceo J.M., Blankenship J.R., Fanning S., Hamaker J.J., Deneault J.S., Smith F.J., Nantel A., Mitchell A.P. (2008). Regulation of the *Candida albicans* cell wall damage response by transcription factor Sko1 and PAS kinase Psk1. Mol. Biol. Cell.

[15] Alonso-Monge R., Román E., Arana D.M., Prieto A.D., Urrialde V., Nombela C., Pla J. (2010). The Sko1 protein represses the yeast-to-hypha transition and regulates the oxidative stress response in *Candida albicans*. Fungal Genet. Biol..

[16] Marotta D.H., Nantel A., Sukala L., Teubl J.R., Rauceo J.M. (2013). Genome-wide transcriptional profiling and enrichment mapping reveal divergent and conserved roles of Sko1 in the *Candida albicans* osmotic stress response. Genomics.

[17] Sellam A., Hoog M.V., Tebbji F., Beaurepaire C., Whiteway M., Nantel A. (2014). Modeling the transcriptional regulatory network that controls the early hypoxic response in *Candida albicans*. Eukaryot. Cell.

[18] Alonso-Monge R., Carvaihlo S., Nombela C., Rial E., Pla J. (2009). The Hog1 MAP kinase controls respiratory metabolism in the fungal pathogen *Candida albicans*. Microbiology.

[19] Proft M., Pascual-Ahuir A., de Nadal E., Arino J., Serrano R., Posas F. (2001). Regulation of the Sko1 transcriptional repressor by the Hog1 MAP kinase in response to osmotic stress. EMBO J..

[20] Proft M., Struhl K. (2002). Hog1 kinase converts the Sko1-Cyc8-Tup1 repressor complex into an activator that recruits SAGA and SWI/SNF in response to osmotic stress. Mol.Cell.

[21] Zapater M., Sohrmann M., Peter M., Posas F., de Nadal E. (2007). Selective requirement for SAGA in Hog1-mediated gene expression depending on the severity of the external osmostress conditions. Mol. Cell. Biol..

[22] Rep M., Proft M., Remize F., Tamas M., Serrano R., Thevelein J.M., Hohmann S. (2001). The Saccharomyces cerevisiae Sko1p transcription factor mediates HOG pathway-dependent osmotic regulation of a set of genes encoding enzymes implicated in protection from oxidative damage. Mol. Microbiol..

[23] Negredo A., Monteoliva L., Gil C., Pla J., Nombela C. (1997). Cloning, analysis and one-step disruption of the *ARG5*,*6* gene of *Candida albicans*. Microbiology.

[24] José C.S., Alonso-Monge R., Pérez-Díaz R.M., Pla J., Nombela C. (1996). The mitogen-activated protein kinase homolog *HOG1* gene controls glycerol accumulation in the pathogenic fungus *Candida albicans*. J. Bacteriol..

[25] Prieto D., Roman E., Alonso-Monge R., Pla J. (2017). Overexpression of the Transcriptional Regulator *WOR1* Increases Susceptibility to Bile Salts and Adhesion to the Mouse Gut Mucosa in *Candida albicans*. Front. Cell. Infect. Microbiol..

[26] Wilson D., Mayer F.L., Miramon P., Citiulo F., Slesiona S., Jacobsen I.D., Hube B. (2014). Distinct roles of *Candida albicans*-specific genes in host-pathogen interactions. Eukaryot. Cell.

[27] Mayer F.L., Wilson D., Hube B. (2013). *Candida albicans* pathogenicity mechanisms. Virulence.

[28] Koffel R., Tiwari R., Falquet L., Schneiter R. (2005). The *Saccharomyces cerevisiae YLL012/YEH1*, *YLR020/YEH2*, and *TGL1* genes encode a novel family of membrane-anchored lipases that are required for steryl ester hydrolysis. Mol. Cell. Biol..

[29] Moyes D.L., Wilson D., Richardson J.P., Mogavero S., Tang S.X., Wernecke J., Hofs S., Gratacap R.L., Robbins J., Runglall M. (2016). Candidalysin is a fungal peptide toxin critical for mucosal infection. Nature.

[30] Richardson J.P., Mogavero S., Moyes D.L., Blagojevic M., Kruger T., Verma A.H., Coleman B.M., Diaz J.D., Schulz D., Ponde N.O. (2018). Processing of Candida albicans Ece1p Is Critical for Candidalysin Maturation and Fungal Virulence. MBio.

[31] Swidergall M., Khalaji M., Solis N.V., Moyes D.L., Drummond R.A., Hube B., Lionakis M.S., Murdoch C., Filler S.G., Naglik J.R. (2019). Candidalysin Is Required for Neutrophil Recruitment and Virulence During Systemic Candida albicans Infection. J. Infect. Dis..

